# ADAMTS13 ameliorates diabetic nephropathy by Nrf2/GPX4/eNOS signaling pathway

**DOI:** 10.1080/0886022X.2026.2646089

**Published:** 2026-03-30

**Authors:** Honghong Wang, Jie Guo, Qingqing Wang, Fanghao Cai, Shan Jiang, Lingyan Fei, Gensheng Zhang, Guizhen Yu, Bingjue Li, Jingyi Zhou, Zheng Li, Fei Han, En Yin Lai, Suhan Zhou

**Affiliations:** a Kidney Disease Center of the First Affiliated Hospital, and Department of Physiology, School of Basic Medical Sciences, Zhejiang University School of Medicine, Hangzhou, China; b Scientific Research Center, The Seventh Affiliated Hospital, Sun Yat-sen University, Shenzhen, China; c Department of Nephrology, Center of Kidney and Urology, The Seventh Affiliated Hospital, Sun Yat-Sen University, Shenzhen, China; d Department of Urology, Pediatric Urolith Center, Children’s Hospital, Zhejiang University School of Medicine, National Clinical Research Center for Child Health, Pediatric Nephrology & Urology Medical Research Center, Hangzhou, China; e Institute of Translational Physiology, Charité–Universitätsmedizin Berlin, Corporate member of Freie Universität Berlin, Humboldt-Universität zu Berlin, and Berlin Institute of Health, Berlin, Germany

**Keywords:** Diabetic nephropathy, Endothelial dysfunction, ADAMTS13, Nrf2, eNOS

## Abstract

Diabetic nephropathy (DN) is a microvascular complication of diabetes mellitus (DM). Accumulated reactive oxygen species (ROS) and oxidative stress-induced ferroptosis and mitochondrial dysfunction play a critical role in the development of DN. The aim of this research was to investigate the protective role and mechanism of ADAMTS13 in regulating oxidative stress-mediated cell death via nuclear factor erythroid 2-related factor 2 (Nrf2) in DN. In this study, DN patients with renal biopsy-confirmed and healthy controls were collected. *In vivo*, DN mice models were established by intraperitoneal injection of streptozotocin, followed by tail vein administration of recombinant human ADAMTS13 (rhADAMTS13). *In vitro,* human glomerular endothelial cells and human umbilical vein endothelial cells were exposed to high glucose. The results demonstrated that serum ADAMTS13 was decreased in DN patients. rhADAMTS13 inhibited ROS generation by activating the Nrf2/GPX4 signaling pathway, thereby inhibiting mitophagy and ferroptosis, ultimately ameliorating renal injury in DN mice. Meanwhile, endothelial nitric oxide synthase (eNOS) phosphorylation was enhanced, which promoted the production of endogenous NO, and then improved vascular endothelial dysfunction. *In vitro*, rhADAMTS13 inhibited the production of ROS in both cytoplasm and mitochondria, while concurrently reducing the release of NO. Our findings suggest that ADAMTS13 may be a potential therapeutic agent for DN through Nrf2/GPX4/eNOS signaling pathway. ADAMTS13 may alleviate DN by inhibiting modulating ferroptosis through the regulation of mitophagy, thereby ameliorating endothelial dysfunction.

## Graphical Abstract

**Figure:**
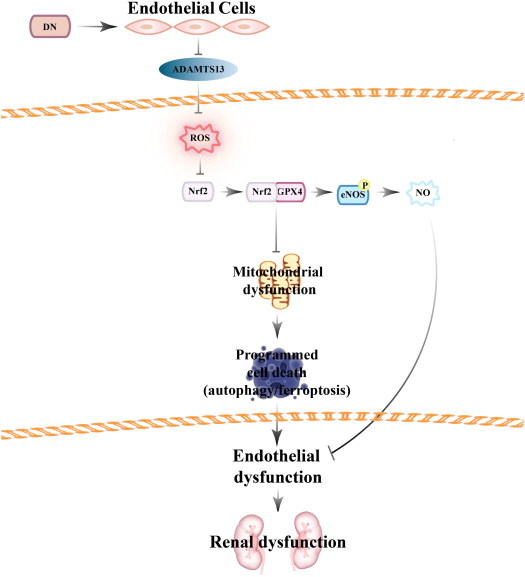

## Introduction

Recent epidemiological data indicate that the global prevalence of diabetes mellitus (DM) has surpassed 463 million cases, with the disease directly causing 1.5 million deaths in 2019, ranking as the ninth leading cause of mortality worldwide. The global prevalence of diabetes is projected to reach 10.2% by 2030 and rise further to 10.9% by 2045 [1]. Diabetes can lead to significant microvascular and macrovascular complications. Based on the DISCOVER study, the global prevalence of diabetic vascular damage was estimated as 12.7% (microvascular) and 18.8% (macrovascular) by 2018 [2]. Macrovascular damage significantly impacts the overall mortality rate of diabetic patients [3]. Diabetic nephropathy (DN), the most common microvascular complication of diabetes, has emerged as a major type of chronic kidney disease (CKD) and a primary cause of end-stage renal disease (ESRD), significantly contributing to the high mortality among diabetic patients [4]. Currently, although lifestyle interventions, blood glucose control and treatments such as sodium-glucose cotransporter-2 (SGLT2) inhibitors can mitigate renal damage to some extent, there remains a lack of effective therapies to halt the progression of DN to ESRD. There is an urgent need to explore novel adjuvant treatment options [5].

Intravascular homeostasis disorder caused by endothelial cell injury, overactivation, and dysfunction has been considered to be the major initiating cause in the pathogenesis of vascular complications in DM [6]. Endothelial dysfunction (ED) induced by hyperglycemia is a critical factor in the development and progression of both microvascular and macrovascular complications in diabetes [7]. Endothelial dysfunction is characterized by the inhibition of endothelial nitric oxide synthase (eNOS), accompanied by reduced production and bioavailability of nitric oxide (NO) [8]. Studies have confirmed that hyperglycemia impairs eNOS function and promotes reactive oxygen species (ROS) generation, leading to decreased NO bioavailability and ultimately disruption of vascular homeostasis. Extensive research has revealed the pathogenic mechanisms by which hyperglycemia induces endothelial dysfunction through oxidative stress, inflammation, and diverse cell death pathways [9]. However, the specific pathways by which diabetes-induced endothelial dysfunction contributes to the progression of DN remain incompletely characterized. An enhanced understanding of endothelial functional alterations in the diabetic condition will be essential for developing effective mechanistically targeted therapies.

Persistent hyperglycemia leads to excessive generation of ROS in both the cytoplasm and mitochondria, promoting the initiation and progression of diabetic vascular complications. Nuclear factor erythroid 2-related factor 2 (Nrf2), a redox-sensitive transcription factor, plays a crucial role in scavenging diabetes-induced free radicals and regulating cellular defense mechanisms against oxidative stress [10]. Impaired Nrf2 activation not only exacerbates oxidative stress and inflammatory responses but also disrupts mitochondrial homeostasis, including biogenesis, proteostasis, and mitophagy, through dysregulation of the dynamin-related protein 1 (DRP1)-dependent mitochondrial fission–fusion equilibrium. Furthermore, mitochondrial dysfunction can alter the redox status of cysteine residues in Kelch-like ECH-associated protein 1 (Keap1), thereby affecting Nrf2 signaling. This vicious cycle between Nrf2 inhibition and mitochondrial dysfunction aggravates renal injury and promotes DN pathogenesis [4,11]. Autophagy is a dynamic process for degrading and recycling damaged macromolecules and organelles to synthesize new cellular components, which is essential for maintaining cellular homeostasis [12]. As a selective form of autophagy, mitophagy specifically removes damaged or excessive mitochondria to maintain mitochondrial dynamics and physiological functions, primarily through the PTEN-induced putative kinase 1 (PINK1)–Parkin regulatory pathway [13]. Accumulating evidence indicates that both autophagy and mitophagy are involved in the development and progression of DN [1,14,15]. A recent study has demonstrated crosstalk between Nrf2 and autophagy in cerebrovascular diseases [16], while other evidence suggests that Nrf2 can directly regulate mitophagy and inhibit ferroptosis in Parkinson’s disease [17]. Ferroptosis represents a recently characterized mode of regulated cell death, characterized by dysregulated iron metabolism, ROS-mediated lipid peroxidation, glutathione depletion, and inactivation of glutathione peroxidase 4 (GPX4) [18,19]. Research indicates that mitophagy-regulated ferroptosis plays a pivotal role in DN progression [20]. Whether Nrf2 exerts protective effects against DN by modulating mitophagy to suppress ferroptosis warrants further investigation. Currently, several Nrf2 activators have shown efficacy in ameliorating diabetes-induced endothlial dysfunction, with a few advancing to clinical trials [2,21]. Therefore, elucidating the pathophysiological role and mechanism of Nrf2 in DN holds significant promise for developing novel therapeutic strategies [1].

Our previous investigation demonstrated that a disintegrin and metalloprotease with thrombospondin type 1 motif member 13 (ADAMTS13) regulate Nrf2 signaling pathway in renal ischemia–reperfusion (I/R) injury [22]. ADAMTS13 is a metalloproteinase produced by endothelial cells, renal tubular epithelial cells, podocytes, and hepatocytes. Animal model studies have demonstrated its aberrant expression in various kidney diseases, suggesting its involvement in renal disease pathogenesis [23]. Research has revealed that ADAMTS13-knockout diabetic mice under diabetic conditions display greater propensity for DN progression [24]. Reduced plasma ADAMTS13 levels correlate with endothelial injury [25], while increased ADAMTS13 activity or exogenous administration of recombinant human ADAMTS13 (rhADAMTS13) exerts protective effects against cerebrovascular diseases, atherosclerosis, and other conditions [26]. The role of ADAMTS13 in DN and its underlying mechanisms remain unclear.

In this study, we aim to investigate: (1) serum ADAMTS13 levels in patients with biopsy-confirmed DN; (2) the protective effects of rhADAMTS13 on renal and aortic tissues in DN models and high glucose-induced human glomerular endothelial cells (HGECs) and human umbilical vein endothelial cells (HUVECs); and (3) the potential mechanisms underlying the therapeutic effects of rhADAMTS13 in DN.

## Materials and methods

### Patient design

Patients were recruited from the Kidney Disease Center of the First Affiliated Hospital, Zhejiang University School of Medicine. We prospectively screened 30 consecutive patients with renal biopsy-confirmed DN who were hospitalized between March 2021 and March 2022, along with 41 healthy living kidney transplant donors (those similar in age and gender) as controls. Clinical data and serum samples were collected from both patients and controls at the time of renal biopsy. This study was approved by the Regional Ethics and Hospital Management Committee of Zhejiang University School of Medicine (Approval No. 0089). The study complied with the Declaration of Helsinki. The requirement for informed consent was waived because this study was based on routinely collected claims data.

Inclusion criteria for DN patients: (1) aged 29–79 years; (2) a history of type 2 diabetes mellitus (T2DM) for more than 5 years; (3) clinical diagnosis of DN, with kidney biopsy findings consistent with DN pathology as the gold standard for diagnosis. Patients met the 2020 American Diabetes Association diagnostic criteria for diabetes, with a urine albumin-to-creatinine ratio (UACR) > 30 mg/g and/or an estimated glomerular filtration rate (eGFR) < 60 mL/(min·1.73 m^2^). Exclusion criteria for DN patients: (1) coexistence of other kidney diseases; (2) evidence of infection during admission; and (3) coexistence of cancer.

Inclusion criteria for healthy controls: (1) aged 33–68 years; (2) normal biomedical parameters within reference ranges. Exclusion criteria for healthy controls: (1) any history of kidney diseases or tumor; (2) any history of infectious diseases.

### Mouse model

All animal procedures and experimental protocols were performed according to the guidelines from Directive 2010/63/EU of the European Parliament on the protection of animals used for scientific purposes or the NIH Guide for the Care and Use of Laboratory Animals. All animal experimental procedures and protocols were reviewed and approved by the Animal Research Ethics Committee of Zhejiang University School of Medicine (Reference Number: No. 2022-337).

**Figure 1.**
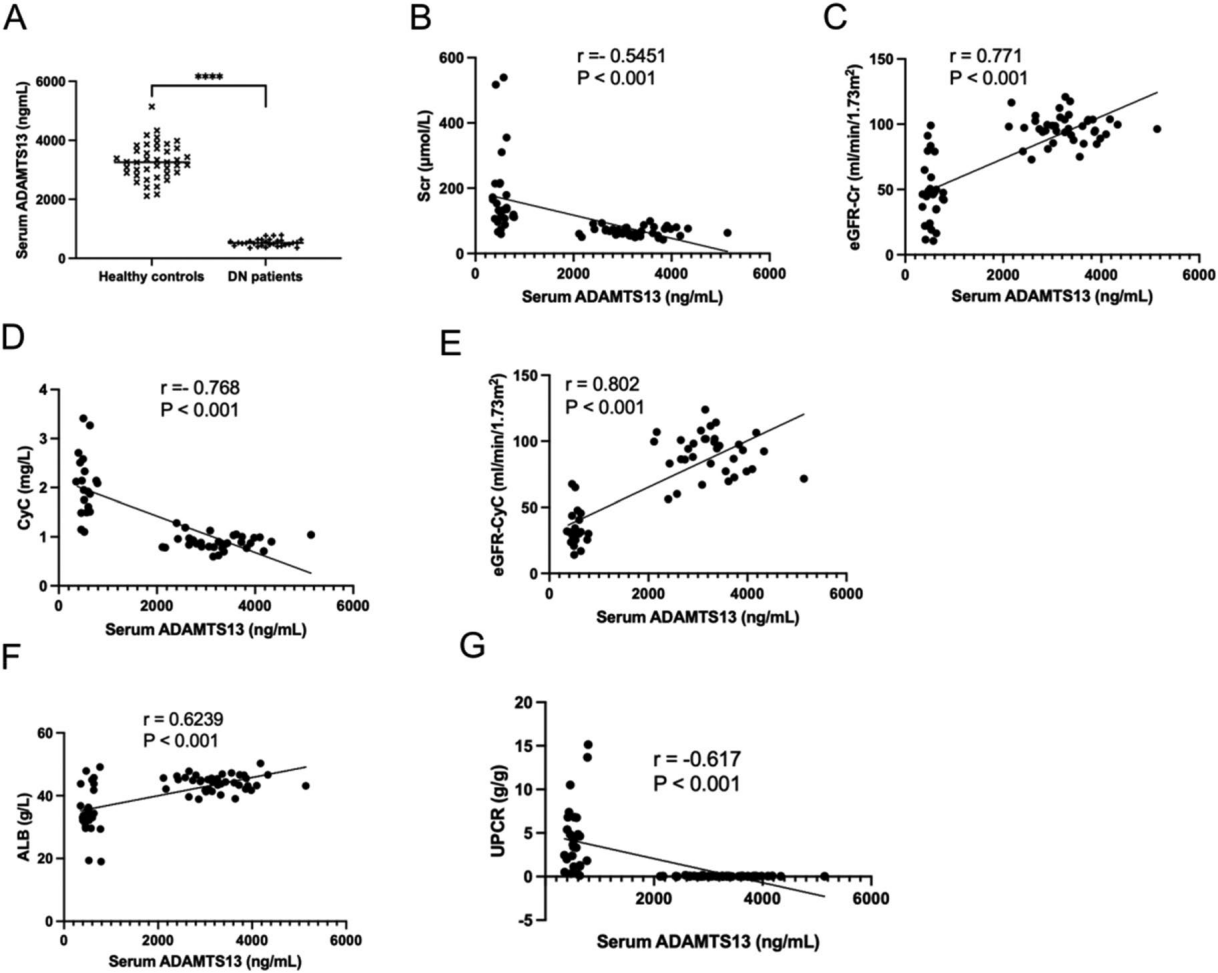
Serum ADAMTS13 was reduced in DN patients and were correlated with renal dysfunction markers. (A) Serum ADAMTS13 in DN patients and healthy controls (healthy controls: *N* = 41, DN patients: *N* = 30). (B) The correlation between serum ADAMTS13 with Scr. (C) The correlation between serum ADAMTS13 with eGFR-Cr. (D) The correlation between serum ADAMTS13 with CyC. (E) The correlation between serum ADAMTS13 with eGFR-CyC. (F) The correlation between serum ADAMTS13 with ALB. (G) The correlation between serum ADAMTS13 with UPCR. Scr: serum creatinine; eGFR: estimated glomerular filtration rate; Cr: creatinine; CyC: cystatin C; ALB: serum albumin; UPCR: urinary protein-to-creatinine ratio. Results were presented as mean ± SEM, ****P < 0.001, two-tailed unpaired Student’s *t*-test, the correlation among the data was evaluated by Pearson’s test.

**Figure 2.**
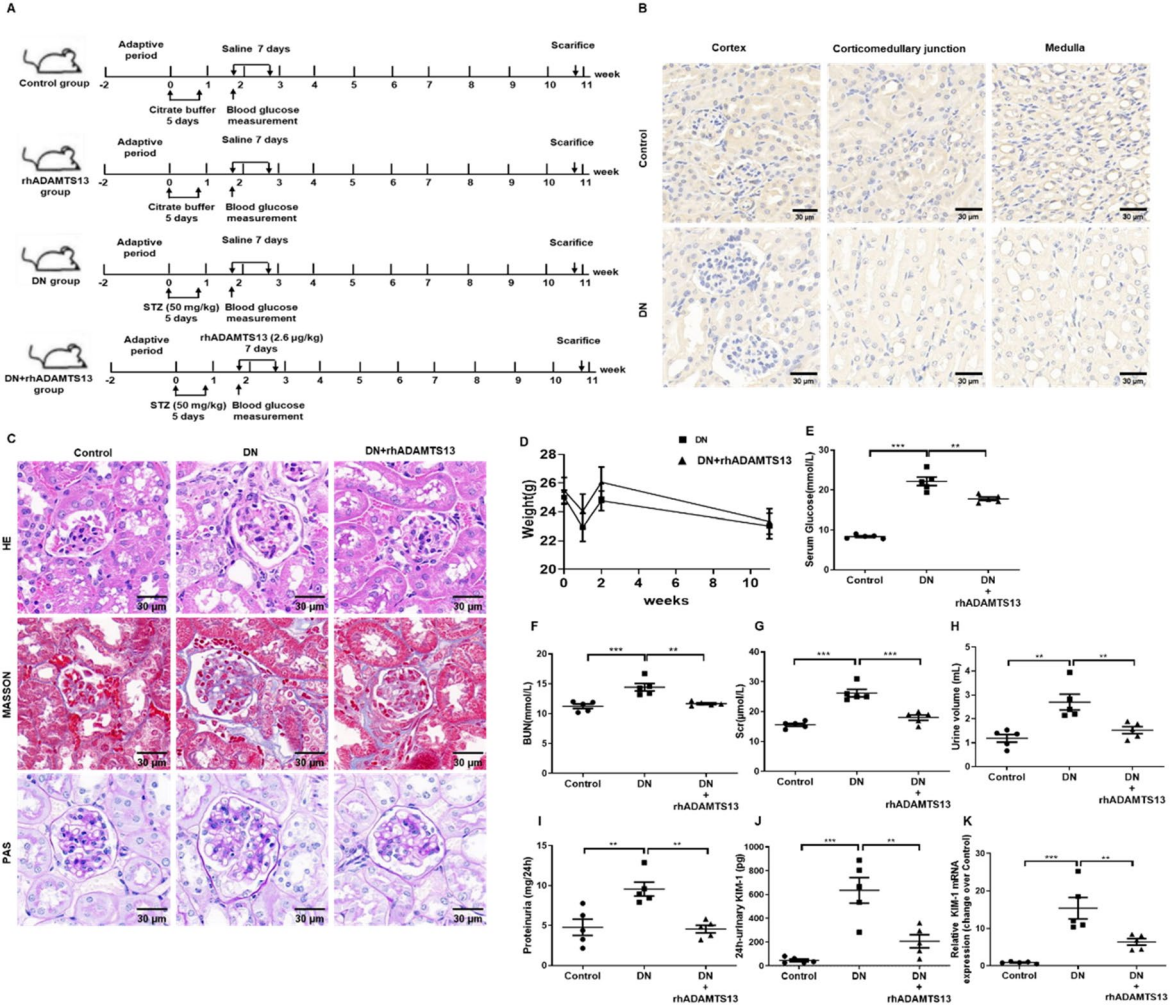
ADAMTS13 maintained renal function and attenuated renal fibrosis *in vivo*. Control mice were treated with vehicle (Control). The STZ-treated mice were infused with vehicle (DN) or rhADAMTS13 (DN + rhADAMTS13). rhADAMTS13 (2.6 ug/kg body weight) was injected into the tail vein daily for the subsequent 7 d. (A) The workflow of animal experiments. (B) Representative renal immunohistochemical staining of ADAMTS13 in the kidney. Scale Bar: 30 μm. (C) Representative renal morphological images of mice were shown. Hematoxylin-Eosin (HE), Masson’s trichrome and Periodic Acid-Schiff (PAS) staining of kidney slices. Scale Bar: 30 μm. (D) Body weight. (E) Serum glucose. (F) BUN. (G) Scr. (H) Urinary volume. (I) Proteinuria. (J) Urinary KIM-1. (K) Renal KIM-1 mRNA expression. ADAMTS13: a disintegrin and metalloprotease with a thrombospondin type 1 motif member 13; DN: diabetic nephropathy; BUN: blood urea nitrogen; Scr: serum creatinine; KIM-1: kidney injury molecule-1. Results were presented as mean ± SEM. *N* = 5, **P < 0.01, ***P < 0.001, one-way ANOVA followed by Tukey’s *post hoc* test.

Since estrogen is the main sex hormone in females and is thought to have renoprotective effects, we chose male mice for the research in this study [27]. Ten- to twelve-week-old C57BL/6 male mice with similar body weights were randomly divided into the following four groups: control group, rhADAMTS13 treatment group, DN group, DN + rhADAMTS13 treatment group, with 5 mice in each group. The mice that received an intraperitoneal injection of citrate buffer (0.1 M, pH 4.5) for five consecutive days were designated the control group. The mice that received an intraperitoneal injection of low-dose streptozotocin (STZ, 50 mg/kg/day) (S0130, Sigma, St. Louis, MO) for five consecutive days were designated the DN group. One week after the last time of STZ injection, blood glucose levels were measured from tail-vein blood samples using the Accu-Chek Aviva glucometer (Roche Diabetes Care, Mannheim, Germany). Hyperglycemia was defined as a fasting blood glucose ≥11.1 mmol/L or random blood glucose ≥16.7 mmol/L. rhADAMTS13 (4245-AD, R&D Systems, Minneapolis, MN) was dissolved in saline and administered to mice via tail vein injections. The mice that were injected with 2.6 μg/kg rhADAMTS13 for seven consecutive days after citrate buffer or STZ injection were designated the rhADAMTS13 group and DN + rhADAMTS13 group. Eight weeks following successful model establishment, mice were anesthetized with 2% isoflurane (R510-22, RWD Life Science Co., Ltd., Shenzhen, China) and euthanized by cervical dislocation, followed by collection of blood, urine, renal tissue, and aortic samples. The animal experimental design is illustrated in Figure 1(A). The animal experimental design is illustrated in Figure 2(A).

### Cell culture and intervention

HGECs were cultured in DMEM (C11885500BT, Gibco, Waltham, MA) supplemented with 10% fetal bovine serum (F2442, Sigma, St. Louis, MO) and 1% penicillin/streptomycin (C100C5, New Cell & Molecular Biotech, Suzhou, China) in the incubator containing 5% CO_2_ at 37 °C. HGECs were starved in DMEM-1% FBS for 12 h prior to treatment and then divided into the following groups: normal glucose (NG, 5.5 mM), high glucose (HG, 35 mM), and high glucose plus rhADAMTS13 (HG + rhADAMTS13, 100 ng/mL) for 24 h [28,29]. To inhibit Nrf2 activity, cells were treated with 20 μM ML385 (HY-100523, MCE, Shanghai, China) for 24 h [30]. To inhibit GPX4 activity, cells were treated with 10 μM Erastin (HY-15763, MCE, Shanghai, China) for 24 h [31]. To inhibit eNOS activity, cells were exposed to 300 μM L-NAME (HY-18729A, MCE, Shanghai, China) for 24 h [32].

HUVECs were cultured in RPMI-1640 (C3010-0500, VivaCell, Shanghai, China) supplemented with 10% FBS and 1% penicillin/streptomycin for serial subcultivation. HUVECs were starved in RPMI-1640-1% FBS for 24 h prior to treatment and then divided into the following groups: NG, HG (44 mM), high glucose plus rhADAMTS13 (HG + rhADAMTS13) for 48 h [33].

### Measurement of renal function

Mice were placed in metabolic cages (Tecniplast, West Chester, PA) for urine collection used for biochemical analysis [34]. We housed well-recovered mice individually in metabolic cages with *ad libitum* access to food and water under controlled environmental conditions (temperature and humidity). Twenty-four-hour urine samples were collected. Proteinuria was assessed using commercial kits (Bethyl Laboratory Inc., Houston, TX). Serum creatinine (Scr) and blood urea nitrogen (BUN) were measured to estimate renal function using commercial kits (048106190, 04460715190, Roche, Mannheim, Germany).

### Measurement of malondialdehyde (MDA) and hydrogen peroxide (H_2_O_2_), enzymatic activity of catalase (CAT)

According to manufacturer’s protocol, the concentration of MDA in renal tissue samples was detected using the Lipid Peroxidation MDA Assay Kit (S0131M, Beyotime, Shanghai, China), the concentration of H_2_O_2_ in urine samples was detected using the Hydrogen Peroxide Assay Kit (S0038, Beyotime, Shanghai, China), the activity of CAT in cell samples was detected using the Catalase Assay Kit (S0051, Beyotime, Shanghai, China).

### Detection of nitric oxide (NO) production

The concentration of NO in urine samples was detected using the Total Nitric Oxide Assay Kit (S0023, Beyotime, Shanghai, China) following the manufacturer’s protocol.

### Enzyme-linked immunosorbent assay (ELISA)

Serum ADAMTS13 (KI-76352, Mskbio, Wuhan, China) (JHN60014, JINHENGNUO, Hangzhou, China) and urinary KIM-1 (JHN80344, JINHENGNUO, Hangzhou, China) were detected using commercial ELISA kits following the manufacturer’s protocols.

### Real-time quantitative polymerase chain reaction (RT-qPCR)

Total RNA was extracted from harvested kidneys (AP-MN-MS-RNA-250, Axygen, San Francisco, CA), and cDNA was synthesized with TaKaRa Reverse Transcription System (RR037A, TaKaRa, Dalian, China). RT-qPCR was tested with SYBR^®^ Premix Ex Taq™ II (RR820A, TaKaRa, Dalian, China) using the CFX96 Real-Time PCR Detection System (BioRad, Hercules, CA) and primers (Table 1). The 2^−ΔΔCT^ method was used to calculate fold changes in target expression levels, with β-actin as the endogenous reference.

**Table 1.** Mice primer sequences.

Primer sequence	Forward (5′–3′)	Reverse (5′–3′)
KIM-1	AAACCAGAGATTCCCACACG	GTCGTGGGTCTTCCTGTAGC
NOX2	TGTGGTTGGGGCTGAATGTC	CTGAGAAAGGAGAGCAGATTTCG
β-actin	AGAGGGAAATCGTGCGTGAC	CAATAGTGATGACCTGGCCGT

### Western blot

Kidney, aorta, and cell samples were homogenized on ice using RIPA lysis buffer (P0017, Beyotime, Shanghai, China) for protein extraction, and detected the protein concentration with BCA Protein Assay Kit (P0010, Beyotime, Shanghai, China). Nuclear and cytoplasmic proteins were extracted using a commercial kit (P0027, Beyotime, Shanghai, China). Equal amounts of protein were loaded on 8–12% sodium dodecyl sulfate-polyacrylamide gels, transferred to polyvinylidene fluoride membrane (PVDF) (Thermo Fisher Scientific, Waltham, MA), and blocked with 5% skim milk powder for 2 h. Membranes were incubated overnight at 4 °C with the following primary antibodies: Beclin-1 (A7353, ABclonal, Wuhan, China); LC3II/I (#4108, 14/16 kDa, CST, Danvers, MA); Nrf2 (ab31163, Abcam, Cambridge, UK); SOD1 (A12537, ABclonal, Wuhan, China); SOD2 (A21805, ABclonal, Wuhan, China); GPX4 (A1933, ABclonal, Wuhan, China); eNOS (#32027, CST, Danvers, MA); p-eNOS (Ser 1177) (AF5812, Beyotime, Shanghai, China); β-actin (AF5002, Beyotime, Shanghai, China); GAPDH (AC001, ABclonal, Wuhan, China); Histone H3 (A2348, ABclonal, Wuhan, China). Thereafter, membranes were washed three times with TBST buffer; they were probed with horseradish-peroxidase-conjugated secondary antibodies ((rabbit, ab6721, Abcam, Cambridge, UK); (mouse, ab6789, Abcam, Cambridge, UK)) for 1 h at room temperature. The bands were visualized using enhanced chemiluminescence reagents (FD8020, Fude Biological Technology Co., Ltd., Hangzhou, China) and imaged with Tanon 5200 Multi Imaging System (Tanon Science & Technology Inc., Shanghai, China). β-actin or GAPDH was used as loading control and protein expression was normalized to β-actin or GAPDH. The protein expression of Control group was used as control to assess the relative density.

### Immunohistochemistry (IHC), hematoxylin–eosin (HE), periodic acid-Schiff (PAS), and Masson’s trichrome staining

Paraffin-embedded formalin-fixed 3 μm sections were stained with HE, PAS, Masson’s trichrome, and IHC for ADAMTS13, PINK1, PARK2/Parkin. The positive expressions of proteins were brownish yellow. Twenty non-overlapping visual fields were continuously selected in each section and detected by Image-Pro Plus Image analysis software [29]. Masson’s trichrome staining is a classical method to detect collagen deposition in kidneys, by which the collagenous fibrosis tissue is stained blue, while the other area is stained red, so renal fibrosis is evaluated by Masson’s trichrome staining [34].

### Measurement of ROS and NO levels

Fluorescent probes DHE and MitoSOX were detected in intracellular and mitochondrial ROS (mtROS), respectively, while DAF-FM DA was detected in intracellular NO [35]. DHE, MitoSOX, and DAF-FM DA (Thermo Fisher Scientific, Waltham, MA) were diluted with serum-free medium. HGECs and HUVECs in six-well plates were washed three times with serum-free medium, then incubated with fluorescent probes for 30 min at 37 °C, respectively. After rinsing twice with serum-free medium, fluorescence intensity was measured using ECLIPSE Ni-U fluorescence microscope (Nikon, Tokyo, Japan). The signal intensity was quantified using the Image J software (Bethesda, MD).

### Immunofluorescence staining

For Nrf2 immunofluorescence staining, HGECs and HUVECs were fixed with 4% paraformaldehyde for 15 min and then rinsed thrice with PBS for 5 min each time. The cells were incubated with the primary antibody Nrf2 overnight 4 °C, followed by incubation with the secondary antibody for 2 h at 4 °C, and the nuclei were then stained with DAPI (1 µg/mL) for 5 min. Images were taken using a fluorescence microscope [36].

### Statistical analysis

All data were presented as mean ± SEM. Statistical analyses were performed using Prism 8 (GraphPad Software, San Diego, CA). Data comparison between two groups was performed by using the two-tailed unpaired Student’s *t*-test. One-way ANOVA followed by Tukey’s *post hoc* test was used to compare the means of >2 independent groups. The correlation among the data was evaluated by Pearson’s test. *p* < 0.05 was considered statistically significant.

## Results

### Serum ADAMTS13 levels were significantly reduced in DN patients and were correlated with renal dysfunction markers

As shown in Table 2, DN patients exhibited clinical characteristics including elevated Scr, cystatin C (CyC), urinary protein-to-creatinine ratio (UPCR), and urinary albumin-to-creatinine ratio (UACR), along with reduced albumin (ALB) levels and reduced eGFR (eGFR-Cr and eGFR-CyC), indicating severe renal impairment and a predisposition to comorbid hypertension. Renal pathological findings in Table 3 revealed severe mesangial proliferation and vascular hyalinosis in DN patients. Moreover, serum ADAMTS13 levels were significantly reduced in DN patients and demonstrated positive correlations with eGFR-Cr, eGFR-CyC, and ALB, while exhibiting negative correlations with Scr, CyC, and UPCR (Figure 1(A–G)). No significant difference in serum ADAMTS13 levels was observed between males and females, regardless of healthy controls or DN patients (Supplementary Figure S1A,B).

**Table 2.** The clinical characteristics of healthy controls and DN patients.

Variables	Healthy controls	DN patients
Age, years	54 ± 1.4	53 ± 2.3
Male, *n* (%)	17 (41.5%)	18 (60%)
Systolic pressure, mmHg	123 ± 3	140 ± 3**
Diastolic pressure, mmHg	75 ± 2	79 ± 2
MAP, mmHg	91 ± 2	99 ± 2*
Hypertension, *n* (%)	6 (14.6%)	24 (80.0%)
<1 year	3	2
1–5 years	3	7
6–10 years	0	9
>10 years	0	6
Serum		
Serum creatinine, µmol/L	67 ± 2.1	176 ± 21.9***
Cystatin C, mg/L	0.89 ± 0.03	2.06 ± 0.11***
Albumin, g/L	44.1 ± 0.4	35.2 ± 1.3***
eGFR-Cr, mL/min	96.9 ± 1.7	46.5 ± 4.3***
eGFR-CyC, mL/min	90.7 ± 2.7	34.2 ± 2.5***
Urine		
UPCR, g/g	0.05 ± 0.01	4.42 ± 0.74***
UACR, g/mol.Cr	1.79 ± 0.28	409.36 ± 146.76***
UIgG/c, g/mol.Cr	0.71 ± 0.13	38.7 ± 15.86***
URBP/c, g/mol.Cr	0.02 ± 0.003	1.10 ± 0.71***
Uβ2-MG/c, g/mol.Cr	0.02 ± 0.002	1.38 ± 0.72***

MAP: mean arterial pressure; eGFR: estimated glomerular filtration rate; Cr: creatinine; CyC: cystatin C; UPCR: urinary protein-to-creatinine ratio; UACR: urinary albumin-to-creatinine ratio; UIgG/c: urinary immunoglobulin G-to-creatinine ratio; URBP/c: urinary retinol binding protein-to-creatinine ratio; Uβ2-MG/c: urinary β2-microglobulin-to-creatinine ratio.

Results were presented as mean ± SEM.

*p < 0.05, **p < 0.01, ***p < 0.001, two-tailed unpaired Student’s *t*-test.

**Table 3.** The renal pathology of healthy controls and DN patients.

Renal pathology	Healthy controls	DN patients
Mesangial proliferation, %		
Mild	24.4	36.3
Moderate	0	18.2
Severe	0	45.5*
Tubular atrophy, %		
0–25%	100	72.7
25–50%	0	27.3
Interstitial inflammation, %		
0–25%	100	63.64
25–50%	0	36.36
Interstitial fibrosis, %		
0–25%	100	63.64
25–50%	0	27.27
>50%	0	9.09
Hyaline degeneration of renal vessels, %		
0–25%	100	9.09***
25–50%	0	0
>50%	0	90.91***

Results were presented as %.

*p < 0.05, ***p < 0.001, two-tailed unpaired Student’s *t*-test.

### Renal ADAMTS13 levels were reduced and exogenous ADAMTS13 supplementation effectively ameliorated renal dysfunction in DN mice

Following five consecutive days of intraperitoneal STZ injections, model mice establishment was confirmed by fasting blood glucose ≥11.1 mmol/L or random blood glucose ≥16.7 mmol/L. The mice subsequently received seven days of tail vein injections of rhADAMTS13. Immunohistochemical analysis revealed reduced renal ADAMTS13 expression in DN mice compared to controls (Figure 2(B)). The body weight of DN mice demonstrated a consistent temporal trend, with no significant difference observed between the DN and DN + rhADAMTS13 groups (Figure 2(D)). Compared to untreated DN mice, rhADAMTS13-treated mice exhibited a significant reduction in blood glucose levels (Figure 2(E)). DN mice exhibited marked renal dysfunction, as evidenced by increased BUN and Scr levels, polyuria, and proteinuria (Figure 2(F–I)). Additionally, urinary KIM-1 concentrations and renal KIM-1 mRNA expression were upregulated, further confirming renal injury (Figure 2(J,K)). The renal pathology of DN in mice was characterized by glomerular hypertrophy and mesangial expansion, confirmed by PAS staining. However, treatment with rhADAMTS13 significantly ameliorated these pathological changes (Figure 2(C)). These findings collectively demonstrated the renal protective effects of ADAMTS13 in DN mice. Furthermore, administration of rhADAMTS13 alone did not significantly affect renal function in mice compared to the control group (Supplementary Figure S2A–D).

### ADAMTS13 attenuated renal oxidative stress and suppressed autophagy in DN mice

**Figure 3.**
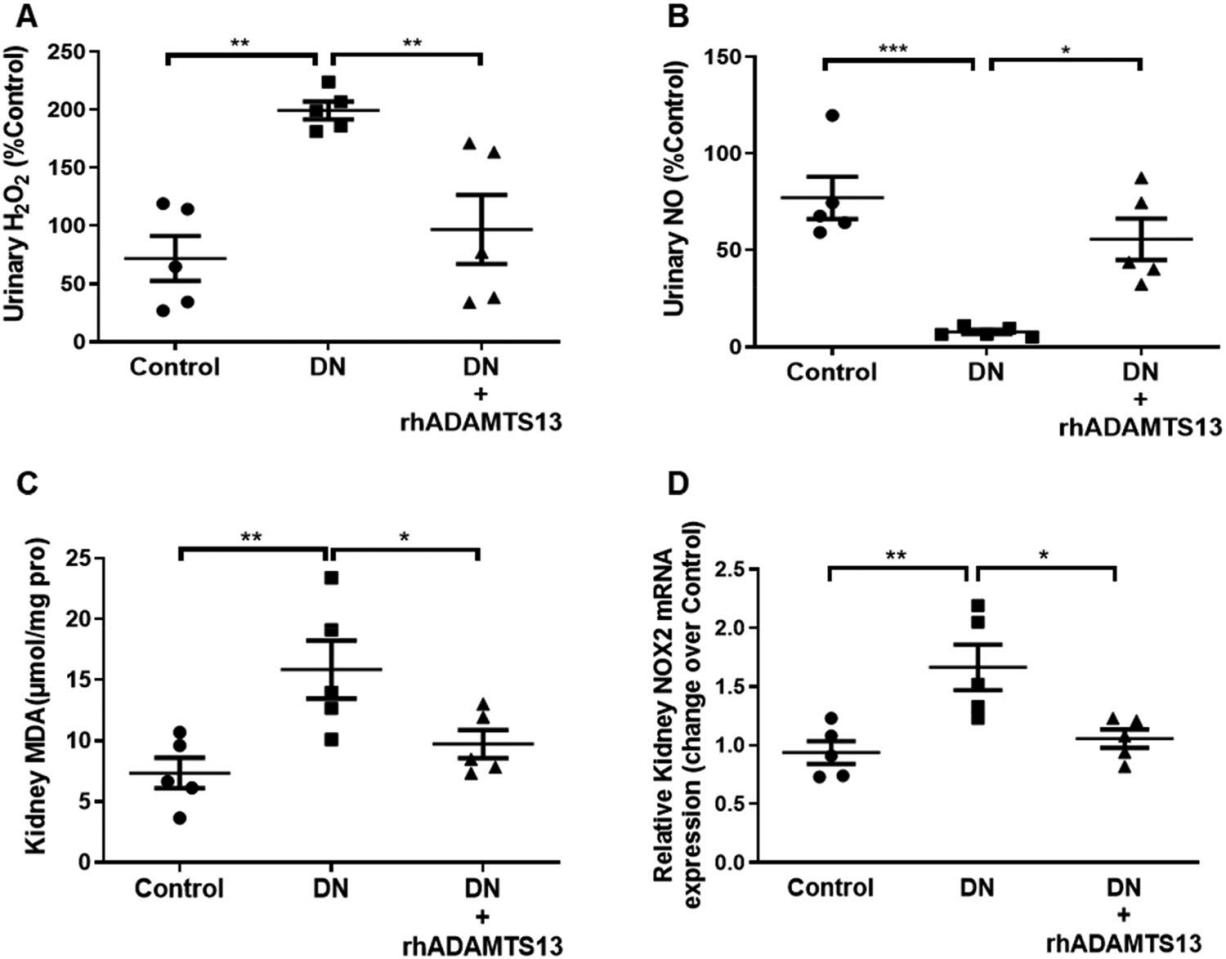
ADAMTS13 attenuated oxidative stress and enhanced NO production in DN mice. (A) Urinary H_2_O_2_. (B) Urinary NO. (C) Kidney MDA. (D) Renal NOX2 mRNA expression. H_2_O_2_: hydrogen peroxide; NO: nitric oxide; MDA: malondialdehyde; NOX: NADPH oxidase. Results were presented as mean ± SEM. *N* = 5, *p < 0.05, ** p < 0.01, ***p < 0.001, one-way ANOVA followed by Tukey’s *post hoc* test.

ROS impairs vascular endothelial function by suppressing NO release in endothelial cells [37]. DN mice exhibited elevated urinary H_2_O_2_ content (Figure 3(A)), increased renal MDA levels and NOX2 mRNA expression (Figure 3(C,D)), accompanied by reduced urinary NO excretion (Figure 3(B)). Treatment with rhADAMTS13 significantly attenuated oxidative stress while promoting the release of NO in DN mice.

**Figure 4.**
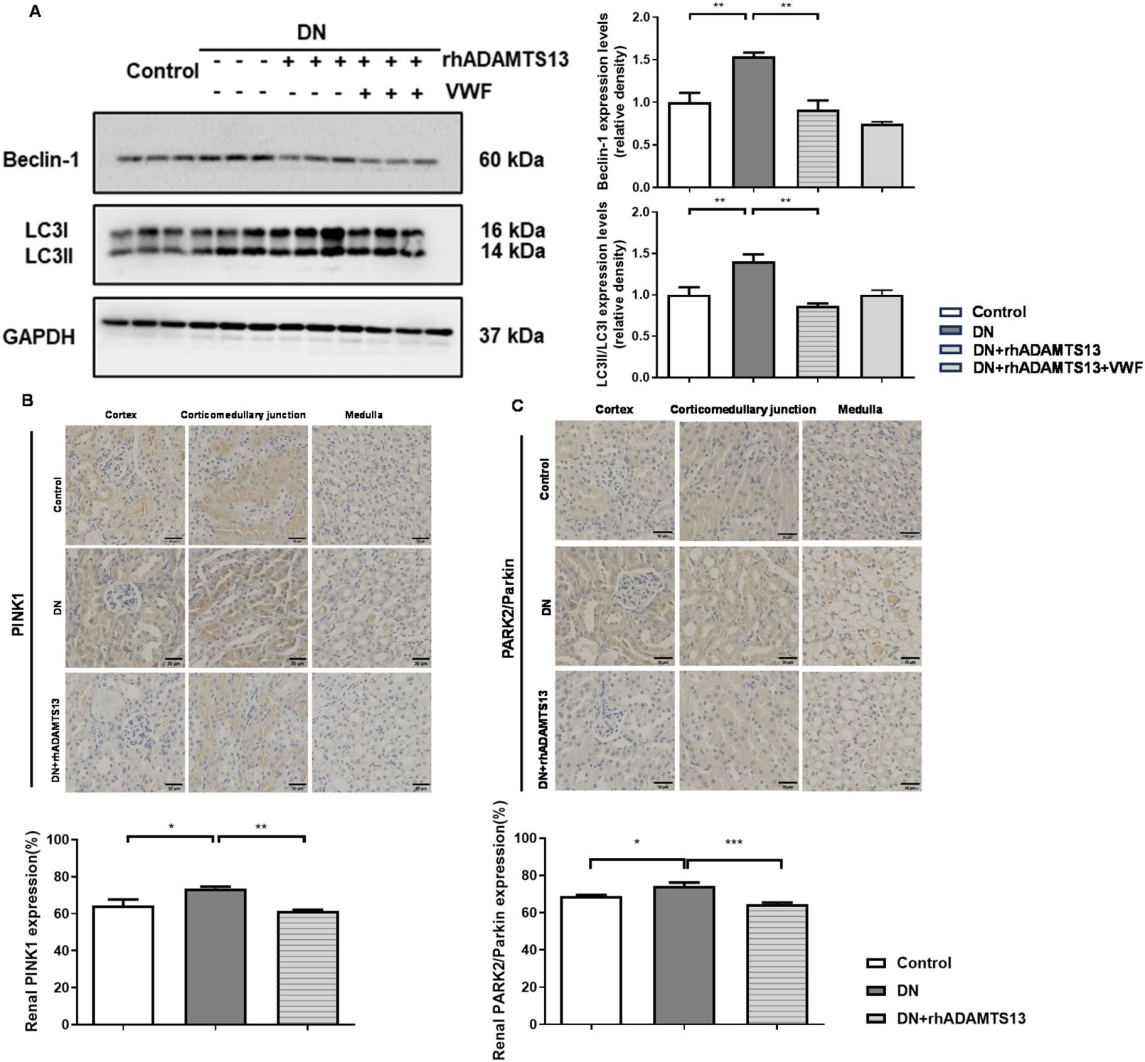
ADAMTS13 exerted partial inhibitory effects on both autophagy and mitophagy in DN mice. (A) Representative images and quantification of Beclin-1 and LC3II/I by western blot analysis (*N* = 3). (B, C) Representative renal immunohistochemical staining of PINK1 and PARK2/Parkin in the kidney and quantitative analysis. Scale bar: 30 μm (*N* = 5). Results were presented as mean ± SEM. *p < 0.05, **p < 0.01, ***p < 0.001, one-way ANOVA followed by Tukey’s *post hoc* test.

Moreover, renal tissues from DN mice showed upregulated Beclin-1 protein expression and elevated LC3II/I ratio, indicating enhanced autophagosome formation. rhADAMTS13 treatment downregulated both Beclin-1 expression and LC3II/I ratio (Figure 4(A)), suggesting partial inhibition of autophagy. Furthermore, the co-administration of VWF did not affect these effects of rhADAMTS13. Immunohistochemical analysis revealed increased PINK1 and PARK2/Parkin expression in DN kidneys, which was suppressed by rhADAMTS13 treatment (Figure 4(B,C)). These results indicated that ADAMTS13 may exert partial inhibitory effects on mitophagy process in DN.

### ADAMTS13 activated Nrf2/GPX4/eNOS pathway, inhibited oxidative stress and ameliorated endothelial dysfunction in DN mice

**Figure 5.**
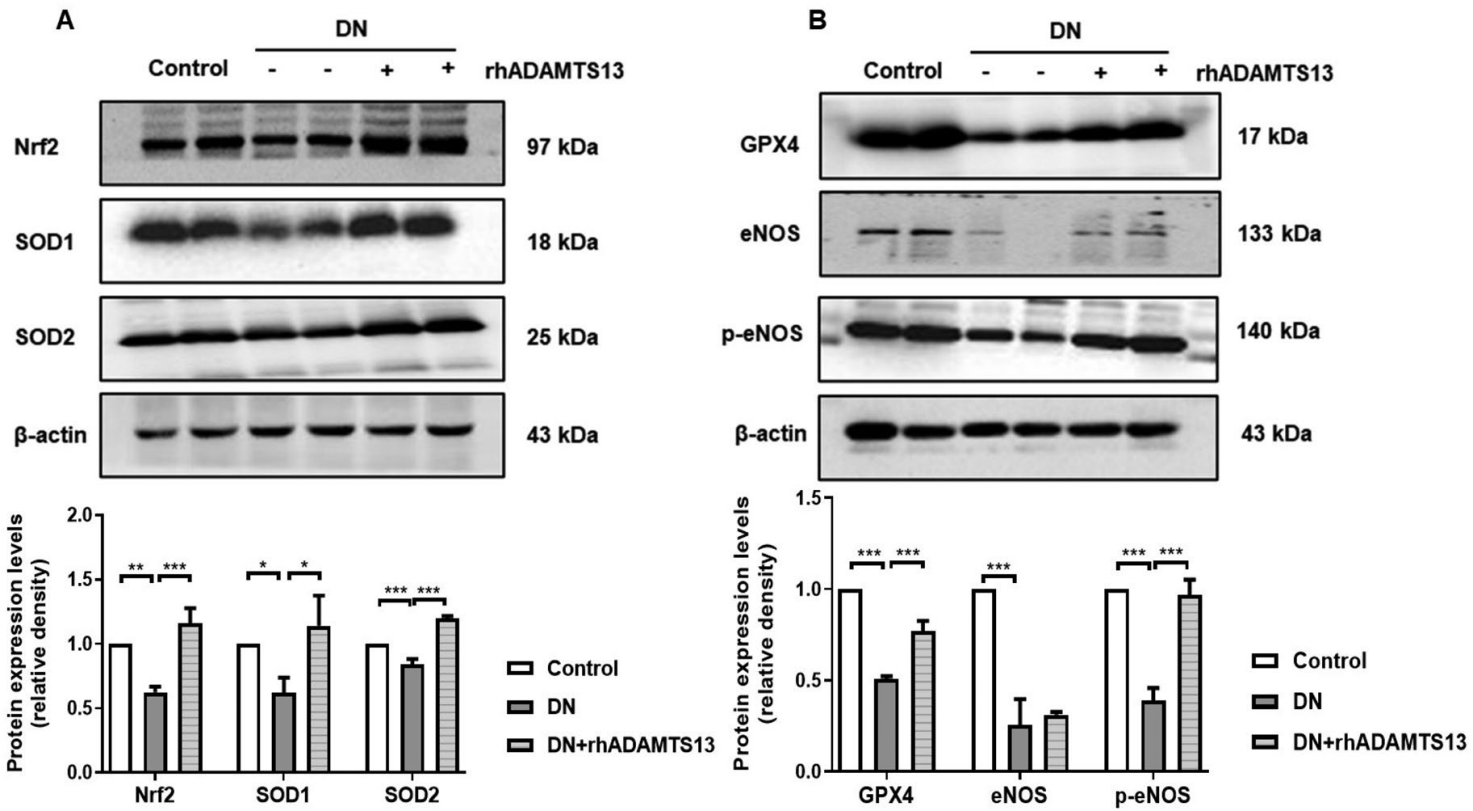
ADAMTS13 activated Nrf2/GPX4/eNOS pathway, inhibited aortic oxidative stress level and ameliorated endothelial dysfunction in DN mice. (A) Representative images and quantification of Nrf2, SOD1, and SOD2 by western blot analysis. (B) Representative images and quantification of GPX4, eNOS, and p-eNOS by western blot analysis. Nrf2: nuclear factor erythroid 2-related factor 2; SOD: superoxide dismutase; GPX4: glutathione peroxidase 4; eNOS: endothelial nitric oxide synthase. Results were presented as mean ± SEM. *N* = 3, *p < 0.05, **p < 0.01, ***p < 0.001, one-way ANOVA followed by Tukey’s *post hoc* test.

Endothelial cell dysfunction is closely associated with the progression of DN [38–40]. Therefore, we investigated the protective role of ADAMTS13 on aortic endothelial injury in DN mice. The expression of Nrf2, SOD1, and SOD2 was significantly reduced in aorta tissues of DN mice, whereas treatment with rhADAMTS13 upregulated these antioxidative proteins and attenuated oxidative stress (Figure 5(A)).

Previous studies have demonstrated that the Nrf2/GPX4 signaling pathway plays a regulatory role in ferroptosis [41,42], while Nrf2 can also modulate eNOS to influence NO production [43,44]. In DN mice, aortic GPX4 expression was reduced, accompanied by suppressed phosphorylation of eNOS. However, rhADAMTS13 administration upregulated GPX4 levels and enhanced the phosphorylation of eNOS (Figure 5(B)). These findings suggested that ADAMTS13 ameliorated vascular oxidative stress by activating the Nrf2/GPX4 pathway while improving endothlial dysfunction through eNOS phosphorylation-mediated mechanism.

### ADAMTS13 induced nuclear translocation of Nrf2 in both high glucose-induced HGECs and HUVECs

**Figure 6.**
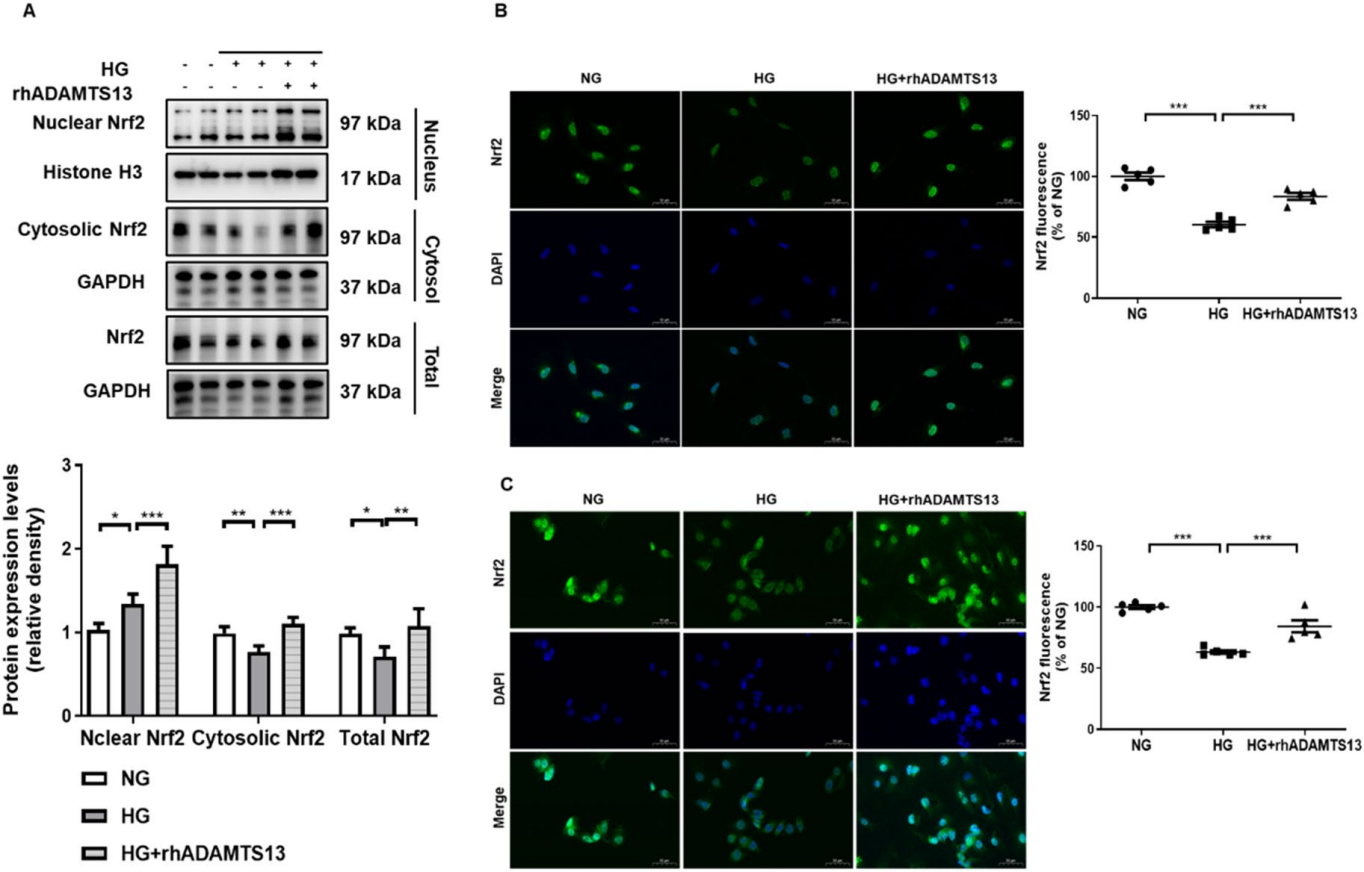
ADAMTS13 induced nuclear translocation of Nrf2 in both high glucose-induced HGECs and HUVECs. (A) Representative images and quantification of nuclear Nrf2, cytosolic Nrf2, and total Nrf2 by western blot analysis in HGECs (*N* = 5). (B, C) Immunofluorescence staining for Nrf2 in HGECs and HUVECs (*N* = 5). Scale bar: 50 μm. HGECs: human glomerular endothelial cells; HUVECs: human umbilical vein endothelial cells. Results were presented as mean ± SEM. *p < 0.05, **p < 0.01, ***p < 0.001, one-way ANOVA followed by Tukey’s *post hoc* test.

Activation of the Nrf2 pathway is dependent upon its nuclear translocation and subsequent induction of antioxidant gene transcription [36]. To explore how ADAMTS13 regulated Nrf2 pathway, we extracted nuclear protein and cytosolic protein in HGECs for the follow-up study, and found that the nuclear expression and cytosolic expression of Nrf2 were increased in rhADAMTS13 treatment group compared to the HG group (Figure 6(A)). Immunofluorescence staining images were then performed to analyze the expression level and location of Nrf2 in HGECs and HUVECs (Figure 6(B,C)). The results showed that in both HGECs and HUVECs, the protein expression and nuclear levels of Nrf2 were significantly increased in the rhADAMTS13 treatment group, and ADAMTS13 could induce Nrf2 nuclear translocation.

### ADAMTS13 inhibited ROS production and ameliorated high glucose-induced endothelial dysfunction by Nrf2/GPX4/eNOS signaling pathway

**Figure 7.**
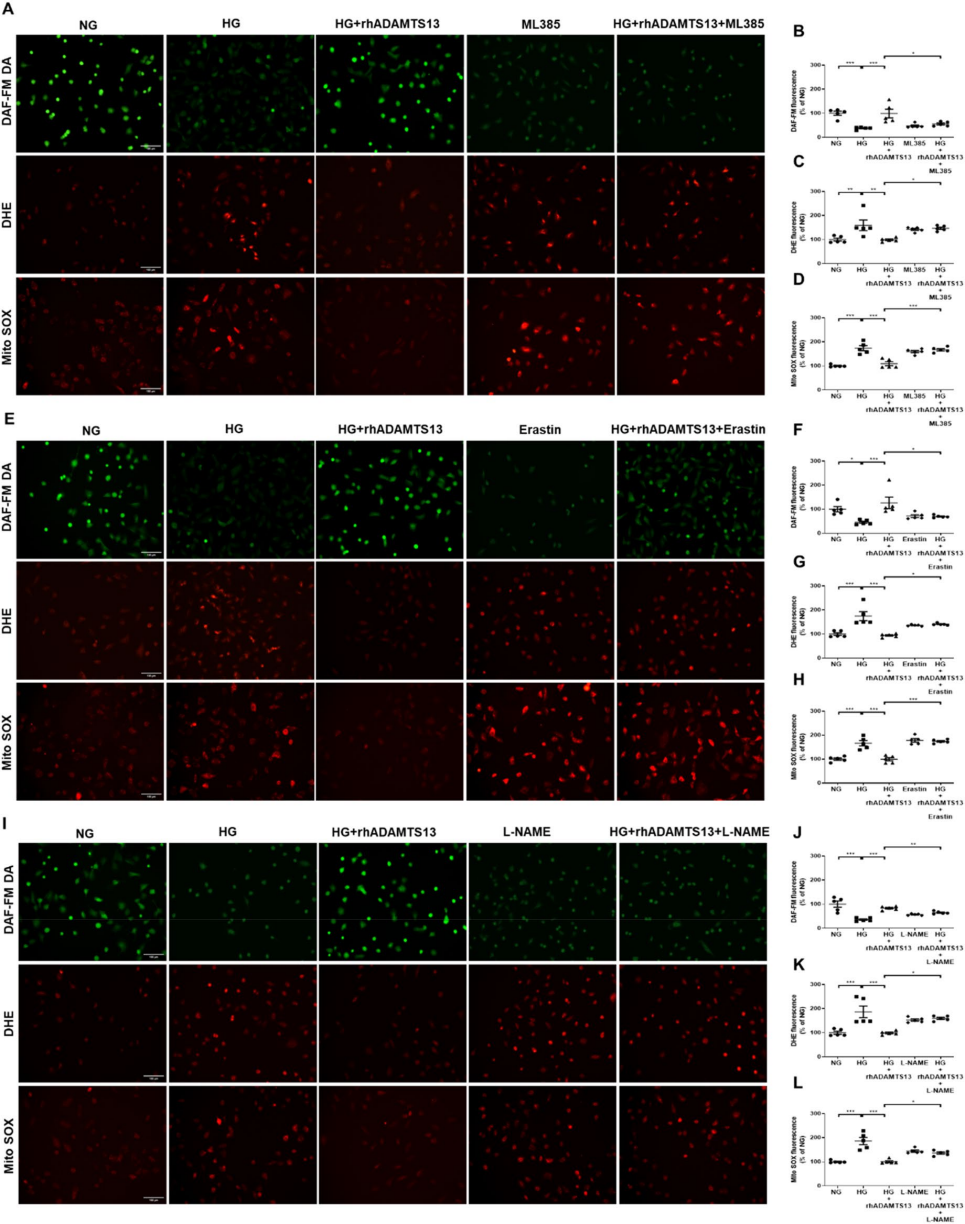
ADAMTS13 promoted NO production and alleviated oxidative stress by Nrf2/GPX4/eNOS signaling pathway in high glucose-induced HGECs. (A, E, and I) Representative images of DAF-FM DA, DHE, and MitoSOX staining were used to assess the production of NO, the cytoplasmic and the mitochondrial ROS generation in HGECs. Scale bar: 100 μm. (B, F, and J) The fluorescence intensity of DAF-FM DA. (C, G, and K) The fluorescence intensity of DHE. (D, H, and L) The fluorescence intensity of MitoSOX. HGECs: human glomerular endothelial cells. Results were presented as mean ± SEM. *N* = 5, *p < 0.05, **p < 0.01, ***p < 0.001, one-way ANOVA followed by Tukey’s *post hoc* test.

Endothelial cell injury leads to vascular endothelial dysfunction. To further investigate the protective effects of ADAMTS13 on endothelial cells, we established an *in vitro* model of HG-induced endothelial cell injury. NO – a crucial vasodilatory paracrine substance released by endothelial cells – is significantly reduced in injured endothelial cells. Quantitative measurement of NO production was done using the DAF-FM DA fluorescent probe, which penetrates cell membranes and generates fluorescence upon binding with NO. High glucose-treated HGECs and HUVECs exhibited decreased fluorescence intensity, indicating reduced NO production and consequent endothelial injury. However, incubation with rhADAMTS13 effectively protected endothelial cells from HG-induced damage (Figure 7(A,B), Supplementary Figure S3A,B).

To evaluate ROS generation in distinct cellular compartments, we performed fluorescent staining using DHE for cytoplasmic superoxide detection and MitoSOX for mitochondrial superoxide detection. High glucose treatment significantly increased superoxide anion levels in both cytoplasm and mitochondria of HGECs and HUVECs (Figure 7(A,C,D), Supplementary Figure S3A,C,D), while simultaneously suppressing CAT activity (Supplementary Figure S3E). The administration of rhADAMTS13 significantly attenuated these oxidative stress injuries. These findings demonstrated that ADAMTS13 exerted potent antioxidant capabilities under hyperglycemic conditions.

To investigate whether Nrf2/GPX4/eNOS signaling is involved in the protective effect of ADAMTS13, we stimulated the HGECs with HG (35 mM) and co-treated with rhADAMTS13 (100 ng/mL), ML385 (20 μM, a Nrf2 inhibitor), Erastin (10 μM, a GPX4 inhibitor), and L-NAME (300 μM, an eNOS inhibitor) for 24 h. As shown in Figure 7(A–D), the DAF-FM DA, DHE, and MitoSOX staining revealed that ML385 significantly attenuated the protective effects of ADAMTS13 against HG-induced endothelial injury and suppressive effect on HG-induced oxidative stress. Similarly, Erastin and L-NAME produced comparable attenuating effects (Figure 7(E–L)). These data suggest that the protective effects of ADAMTS13 against HG induced endothelial injury and oxidative stress are mediated through the Nrf2/GPX4/eNOS signaling pathways.

## Discussion

**Figure 8.**
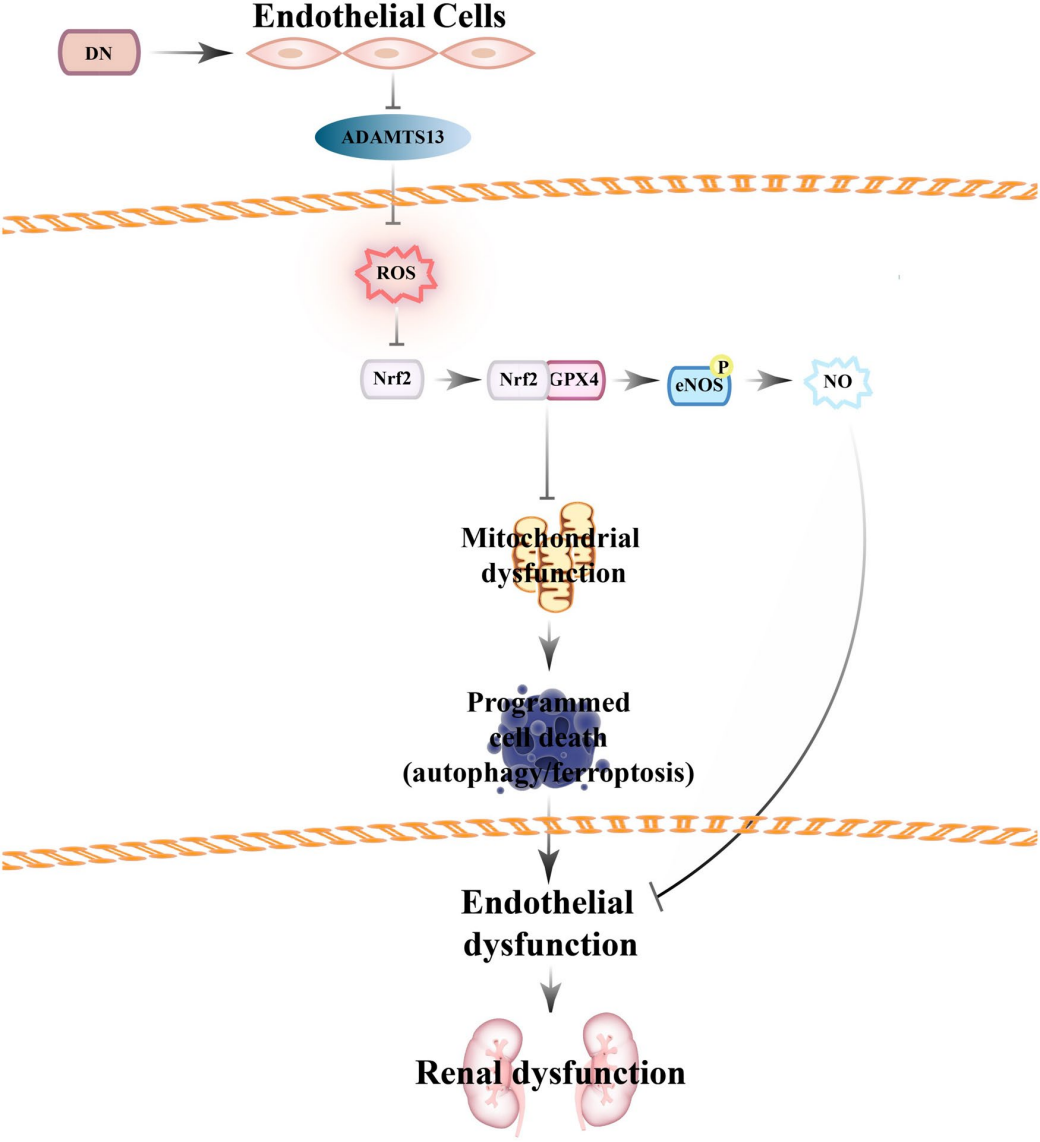
Protective effect of ADAMTS13 on endothelial dysfunction induced by diabetic nephropathy.

In this study, we observed reduced ADAMTS13 levels in both serum samples from DN patients and renal tissues from DN mice, accompanied by marked glucose metabolism abnormalities and renal dysfunction. Administration of rhADAMTS13 *in vivo* effectively ameliorated renal injury and blood glucose levels in DN mice without affecting body weight. Mechanistically, rhADAMTS13 exerted renal protection through a dual pathway: on the one hand, rhADAMTS13 inhibited oxidative stress and mitophagy by activating the Nrf2 signaling pathway, thereby protecting renal cells from autophagy and ferroptosis in DN mice; on the other hand, rhADAMTS13 promoted the phosphorylation of eNOS and the release of NO in endothelial cells, ultimately improving vascular endothelial dysfunction in DN mice. These findings demonstrated that ADAMTS13 exerted a protective role in preserving renal function under diabetic conditions, highlighting its therapeutic potential for DN (Figure 8).

ADAMTS13 is synthesized in hepatic stellate cells, endothelial cells, tubular epithelial cells, and podocytes [45,46]. Previous studies have reported decreased serum ADAMTS13 levels in animal models of I/R kidney injury and DN [22]. Transcriptomic analyses of chronic glomerular diseases – including Alport syndrome, FSGS, and membranous nephropathy – further identified ADAMTS13 as one of the most downregulated genes [47]. Endothelial cells and podocytes represent two principal cell types involved in the pathogenesis of DN. Consistent with prior findings, our study demonstrated significantly reduced ADAMTS13 levels in the serum of DN patients, showing positive correlations with eGFR-Cr, eGFR-CyC, and ALB, while showing negative correlations with Scr, CyC, and UPCR. Moreover, ADAMTS13 expression was reduced in the kidneys of DN animal models, accompanied by increased BUN, Scr, and KIM-1 levels, suggesting a potential role of ADAMTS13 in DN pathogenesis. Importantly, administration of rhADAMTS13 effectively reversed these pathological alterations and ameliorated renal dysfunction in DN mice.

Mitochondria are indispensable intracellular organelles responsible for ATP production, with key functions including maintenance of redox and iron homeostasis, regulation of cell death, inflammation responses, and intracellular calcium balance. Mitochondrial dysfunction contributes to tissue damage and subsequent organ failure [48]. The kidney is the second most mitochondria-rich organ, surpassed only by the heart, making healthy mitochondria crucial for normal renal function [49]. Both autophagy and mitophagy, particularly through the Beclin-1/LC3 and PINK1/Parkin pathways, have been implicated in the pathogenesis of DN [50]. Beyond our expectation, our study revealed that treatment with rhADAMTS13 not only suppressed the elevated levels of Beclin-1 and LC3II/I in renal tissues of DN mice, but also downregulated PINK and Parkin expression in renal tissues of DN mice. This finding suggested that ADAMTS13 may modulate the expression of autophagy- and mitophagy-related proteins. Von Willebrand factor (VWF) is the activation substrate of ADAMTS13, and ADAMTS13 regulates platelet tethering through VWF [34]. Our previous studies demonstrated that VWF promotes the renal tubular fibrosis in CKD mice following rhADAMTS13 treatment [34]. However, rhADAMTS13 alleviates renal fibrosis in UUO mice by inhibiting the TGF-β1/Smad pathway, and this process is not affected by VWF [29]. These findings indicate that the regulatory effect of VWF on ADAMTS13 differs across kidney disease models. In this study, ADAMTS13 alleviated renal damage in DN mice by inhibiting autophagy, and it was not affected by VWF. The role of autophagy in DN pathogenesis remains controversial. While our data indicated that autophagy inhibition exerted renal protective effects in DN, contrasting evidence suggested that autophagy activation may protect endothelial cell function in DN [51]. The precise mechanisms underlying the relationship between autophagy and DN pathogenesis require further elucidation.

Hyperglycemia and other diabetes-related metabolites lead to excessive ROS production in cells [52]. Hydrogen peroxide and lipid peroxidation products such as MDA have been quantified to evaluate oxidative stress levels in diabetic patients. Although mitochondrial oxidative metabolism remains the primary source of superoxide overproduction in hyperglycemic conditions, other oxidoreductases including NADPH oxidase (NOX), cyclooxygenase (COX), and myeloperoxidase (MPO) also significantly contribute to ROS generation in DN tissues [53]. NADPH oxidase 2 (NOX2), an isoform of the NOX enzyme complex that catalyzes electron transfer across biological membranes to generate ROS, is significantly upregulated during myocardial I/R injury [54]. This research demonstrated that rhADAMTS13 inhibited urinary H_2_O_2_, renal MDA and NOX2 levels, indicating the antioxidant capacity of ADAMTS13 in DN.

Continuous high glucose exposure in vascular endothelial cells induces ROS production and activates oxidative stress pathways, leading to endothelial cell injury [55]. Redox imbalance, dysregulated hypoxia-inducible factor (HIF) signaling and mitochondrial dysfunction contribute to diabetes-related cardiovascular tissue damage, while antioxidants can prevent these diabetic vascular complications [1,56]. Both macrovascular and microvascular complications significantly impact the quality of life and prognosis of diabetic patients. Nrf2 is a key antioxidant transcription factor and is involved in the regulation of oxidative stress. In the cell nucleus, Nrf2 binds to antioxidant response elements (AREs) to upregulate the expression of genes encoding phase II detoxification enzymes such as SOD. GPX4, a crucial antioxidant enzyme, reduces lipid peroxides in either non-coupled or lipid-bound forms, serving as a central defender against ferroptosis [54].

Activation of the Nrf2/GPX4 signaling pathway modulates ROS generation, enhances antioxidant enzyme activity and mitigates ferroptosis [57,58]. Endothelial nitric oxide synthase (eNOS) dysfunction is a pathophysiological hallmark of vascular endothelial impairment. In the presence of cofactors such as tetrahydrobiopterin (BH4), eNOS catalyzes L-arginine oxidation to generate NO and L-citrulline [59]. Endothelial function impairment is characterized by reduced NO bioavailability resulting from eNOS dysfunction, with progressive vasculopathy being associated with NO deficiency and increased ROS generation [2,10]. This study demonstrated that rhADAMTS13 treatment activated the Nrf2/GPX4 pathway in DN mice, suppressing oxidative stress and lipid peroxidation while promoting eNOS phosphorylation and increasing NO level. The balance between mtROS production and elimination is essential for proper mitochondrial function. Excessive mtROS accumulation resulting from increased generation or impaired antioxidant defenses may induce mitochondrial dysfunction, leading to cell death, inflammation, and renal injury. The research demonstrated that rhADAMTS13 treatment reduced both cytosolic and mitochondrial ROS production in HG-stimulated HGECs and HUVECs, while enhancing CAT activity and promoting NO release. These findings collectively confirmed that ADAMTS13 suppressed oxidative stress in DN mice, thereby ameliorating endothelial cell injury and vascular endothelial dysfunction.

This study has several limitations that should be acknowledged. First, endothelial cell-specific ADAMTS13 knockout mice were not used to validate the protective role of ADAMTS13 in renal function impairment in DN. Second, although rhADAMTS13 demonstrated protective effects in DN animal model, its therapeutic efficacy in human DN patients required further exploration due to the complexity and heterogeneity of individual responses. Despite these limitations, our study provided compelling experimental evidence supporting the protective role of ADAMTS13 in DN and elucidated its underlying mechanisms. These findings suggested ADAMTS13 as a potential therapeutic target for DN treatment.

## Supplementary Material

Supp figures.docx

## Data Availability

The published preprint is available on request from the corresponding author.
