# Supplementary material for: ADAMTS13 ameliorates diabetic nephropathy by Nrf2/GPX4/eNOS signaling pathway
Source: Ren Fail. 2026 Mar 30;48(1):2646089. doi: 10.1080/0886022X.2026.2646089 (PMC13037144; doi:10.1080/0886022X.2026.2646089)

**ADAMTS13 ameliorates diabetic nephropathy by Nrf2/GPX4/eNOS signaling pathway**

Honghong Wang ^a, 1^,Jie Guo ^a, 1^, Qingqing Wang ^b, 1^, Fanghao Cai ^a^, Shan Jiang ^c^, Lingyan Fei ^c^, Gensheng Zhang ^d^, Guizhen Yu ^a^, Bingjue Li ^a^, Jingyi Zhou ^a^, Zheng Li ^a^, Fei Han ^a^，En Yin Lai ^a, e, *^, Suhan Zhou^a, *^

^a^ Kidney Disease Center of the First Affiliated Hospital, and Department of Physiology, School of Basic Medical Sciences, Zhejiang University School of Medicine, Hangzhou 310003, China

^b^ Scientific Research Center, the Seventh Affiliated Hospital, Sun Yat-sen University, Shenzhen 518107, China.

^c^ Department of Nephrology, Center of Kidney and Urology, the Seventh Affiliated Hospital, Sun Yat-Sen University, Shenzhen 518107, China.

^d^ Department of Urology, Pediatric urolith Center, Children’s Hospital, Zhejiang university School of Medicine, National Clinical Research Center for Child Health, Pediatric Nephrology & Urology Medical Research Center, Hangzhou 310053, China

^e^ Charité–Universitätsmedizin Berlin, Corporate member of Freie Universität Berlin, Humboldt‐Universität zu Berlin, and Berlin Institute of Health, Institute of Translational Physiology, Berlin, Germany

**Supplementary Figure S1.** Serum ADAMTS13 levels in females and males across healthy controls and DN patients**.** (A) Serum ADAMTS13 levels in healthy controls (Female: N=24, male: N=17). (B) Serum ADAMTS13 levels in DN patients (Female: N=12, male: N=18). Results were presented as mean ± SEM, two-tailed unpaired Student’s *t*-test.

**
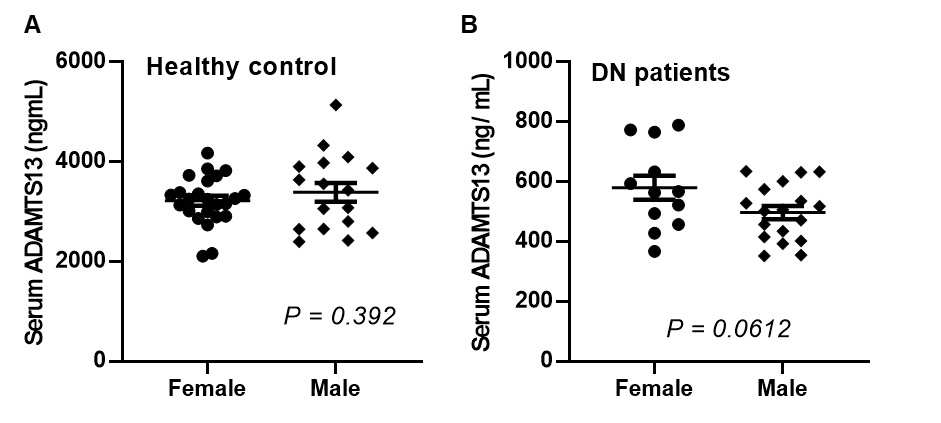
**

**Supplementary Figure S2.** Administration of rhADAMTS13 itself did not significantly affect renal function in mice**.** Mice were treated with vehicle (Control group) or rhADAMTS13 (rhADAMTS13 group). (A) BUN. (B) Scr. (C) Urinary volume. (D) Proteinuria. Results were presented as mean ± SEM. N=5, two-tailed unpaired Student’s *t*-test.


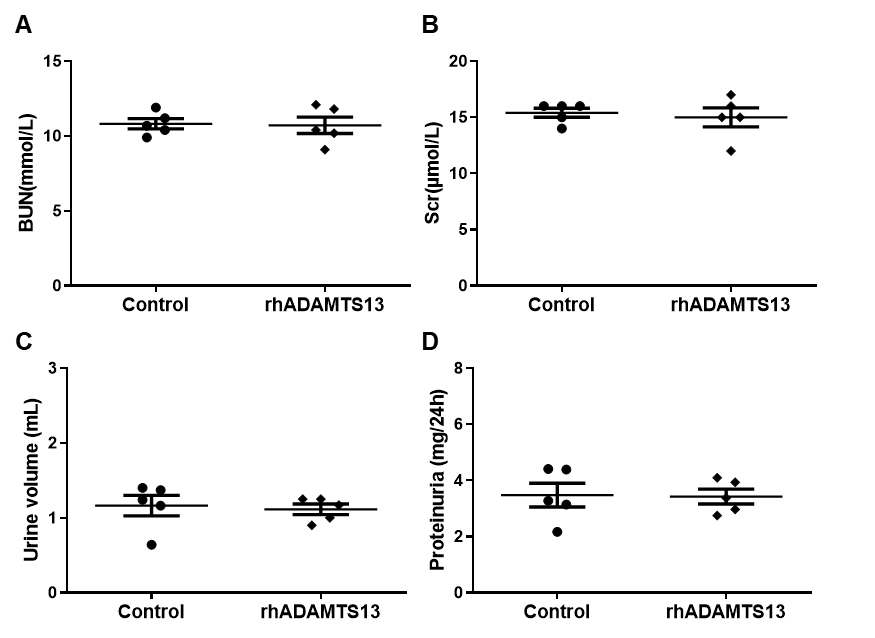


**Supplementary Figure S3. ADAMTS13 promoted NO production and alleviated oxidative stress in high glucose-induced HUVECs.** (A) Representative images of DAF-FM DA, DHE and MitoSOX staining were used to assess the production of NO, the cytoplasmic and the mitochondrial ROS generation in HUVECs. Scale Bar: 100 μm. (B), (C) and (D) the fluorescence intensity of DAF-FM DA, DHE and MitoSOX. (E) Cell catalase (CAT). NO, nitric oxide; HUVECs, human umbilical vein endothelial cells; ROS, reactive oxygen species. Results were presented as mean ± SEM. N=5, **P < 0.05*, ***P < 0.01*, ****P < 0.001*, one-way ANOVA followed by Tukey’s post hoc test.


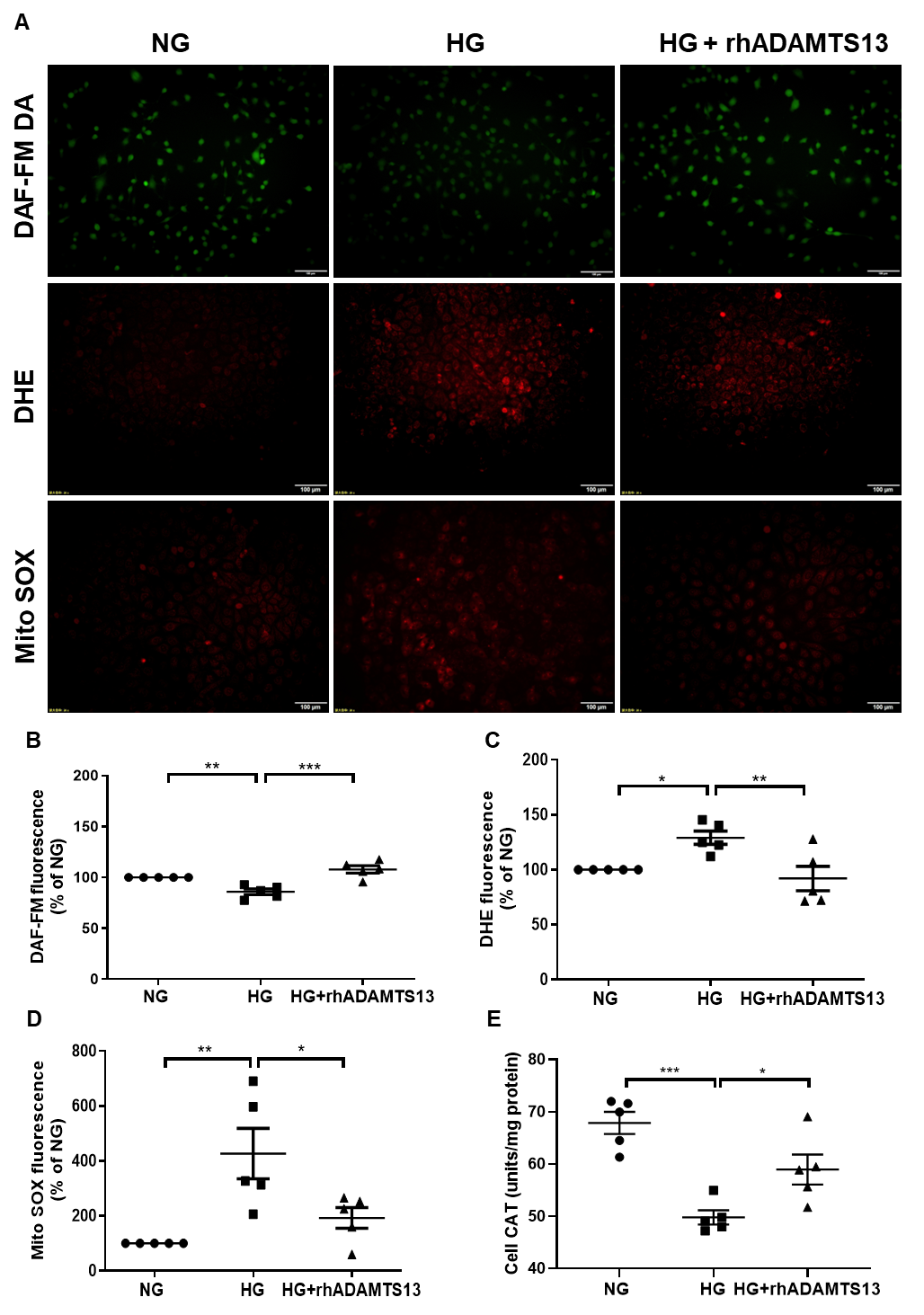

Supplement: Supp figures.docx [file IRNF_A_2646089_SM4831.docx]
